# Development of Synthetic Modulators of PPARs: Current Challenges and Future Opportunities

**DOI:** 10.1155/2008/786359

**Published:** 2009-03-23

**Authors:** Jane A. Pinaire, Anne Reifel Miller, Francine M. Gregoire

**Affiliations:** ^1^Lilly Research Laboratories, Lilly Corporate Center, Indianapolis, IN 46285, USA; ^2^Department of Biology, Metabolex, Inc., 3876 Bay Center Place, CA 94545, USA

The cloning of the mouse PPAR alpha gene in 1990 by Issemann and Green [1] stimulated intense interest in this family of nuclear receptors, and research efforts over the next decade established important roles for the PPAR isotypes in glucose and lipoprotein metabolism, inflammation, and atherosclerosis. Though the fibrates (PPAR-*α* agonists) had been used for the treatment of dyslipidemia for nearly 40 years, the discovery of the insulin-sensitizing effects of PPAR-*γ* agonists brought about the development and commercialization of the thiazolidinedione (TZD) class of oral anti-hyperglycemic medications: troglitazone, approved by the FDA in 1997, and pioglitazone and rosiglitazone, both approved by the FDA in 1999. The withdrawal of troglitazone from the market in 2000 was an early indicator of the potential safety issues of PPAR drugs. Even so, the clinical use of fibrates and TZDs has allowed for a better understanding of the safety profiles and safety issues of PPAR-*α* and PPAR-*γ* agonists [2, 3].

At the beginning of this decade, many pharmaceutical companies had development programs focused on delivering “new and improved” PPAR agonists to the market. For example, several programs were developing PPAR-*α*/*γ* dual agonists (i.e., glitazars) for the treatment of type 2 diabetes. Although preliminary data from various PPAR-*α*/*γ* dual agonist research programs was promising, nearly all of these research programs were discontinued due to safety issues identified during clinical testing and/or during preclinical testing [4–6]. More recently, the highly publicized (and controversial) meta-analysis of rosiglitazone reported by Nissen and Wolski [7] called into question the safety of the TZDs and prompted changes to the labels of both rosiglitazone and pioglitazone.

And yet, the promise of the therapeutic potential of PPAR drugs remains. A PubMed search using the term “peroxisome proliferator-activated receptor” yielded 1578 manuscripts (161 of which were review articles) between January 1 and December 1, 2008. In addition to the roles of the PPAR isotypes in lipid/lipoprotein/glucose metabolism, additional roles in diverse physiological processes and disease states are currently being investigated. As additional functions are identified; the PPARs will continue to be important molecular targets for identifying ligands (drugs) with potential applications to reproduction and fertility, normal development; function of the reproductive, gastrointestinal, respiratory, and central nervous systems; skin biology and wound healing; and cell cycle control and cancer.

Reports highlighting both challenges and opportunities in PPAR drug development are included in this special issue. For example, edema, weight gain, and a reduction in bone mass (particularly in women) are challenges limiting the clinical utility of the currently marketed TZDs. The renal and vascular mechanisms of TZD-induced fluid retention are reviewed by Yang and Soodvilai, and the recent clinical data describing the effects of TZDs on bone are reviewed by Schwartz. In contrast, several reports highlight opportunities in PPAR drug development. Deeg and Tan compare the effects of rosiglitazone and pioglitazone on lipids, lipoproteins, and apolipoproteins as reported in head-to-head, randomized clinical studies. Two papers describe the effects of PPARs/PPAR ligands on immune/inflammatory responses; Fernandez reviews the roles of the PPARs in modulating the immune/inflammatory response in atherosclerosis, while Yamashita reviews the receptor-independent effects of PPAR-*α* and PPAR-*γ* ligands on cysteinyl leukotriene production in mast cells as it relates to the development of potential anti-asthma medications. Technological and methodological approaches that may prove useful in the identification and assessment of new PPAR drugs are also reported. Clarke et al. describe an approach used to determine the species differences in plasma protein binding to MBX-102, a novel PPAR-*γ* agonist currently in Phase 2 clinical development, and the corresponding differences in PPAR-*γ* activation across species. Cho et al. review the role of PPARs in metabolic disorders as well as various strategies and technologies used in the identification and assessment of PPAR drugs. Finally, Miyachi and Hashimoto describe the synthesis and SAR of subtype-specific PPAR agonists derived from a single 3,4-disubstituted phenylpropanoic acid “versatile template” scaffold, and Higgins and Mantzoros review the development and safety profile of INT-131, a potent non-TZD selective PPAR modulator (SPPARM) currently in Phase 2 clinical development.

In conclusion, though the full therapeutic potential of PPARs has yet to be realized, and serious safety issues are associated with the currently marketed PPAR drugs (PPAR-*α* and PPAR-*γ*), there remains intense interest in exploring new physiological roles of the PPARs and in the identification of new and improved PPAR agonists drugs.

*Jane A. Pinaire* *Jane A. Pinaire* *Anne Reifel Miller* *Anne Reifel Miller* *Francine M. Gregoire* *Francine M. Gregoire*

## References

[1] Issemann I, Green S (1990). Activation of a member of the steroid hormone receptor superfamily by peroxisome proliferators. *Nature*.

[2] Rubenstrunk A, Hanf R, Hum DW, Fruchart J-C, Staels B (2007). Safety issues and prospects for future generations of PPAR modulators. *Biochimica et Biophysica Acta*.

[3] Shearer BG, Billin AN (2007). The next generation of PPAR drugs: do we have the tools to find them?. *Biochimica et Biophysica Acta*.

[4] Fiévet C, Fruchart J-C, Staels B (2006). PPAR*α* and PPAR*γ* dual agonists for the treatment of type 2 diabetes and the metabolic syndrome. *Current Opinion in Pharmacology*.

[5] Waites CR, Dominick MA, Sanderson TP, Schilling BE (2007). Nonclinical safety evaluation of muraglitazar, a novel PPAR*α*/*γ* agonist. *Toxicological Sciences*.

[6] Balakumar P, Rose M, Ganti SS, Krishan P, Singh M (2007). PPAR dual agonists: are they opening Pandora's Box?. *Pharmacological Research*.

[7] Nissen SE, Wolski K (2007). Effect of rosiglitazone on the risk of myocardial infarction and death from cardiovascular causes. *The New England Journal of Medicine*.

